# Heterogeneity in African savanna elephant distributions and their impacts on trees in Kruger National Park, South Africa

**DOI:** 10.1002/ece3.7465

**Published:** 2021-04-06

**Authors:** Joel O. Abraham, Emily R. Goldberg, Judith Botha, A. Carla Staver

**Affiliations:** ^1^ Department of Ecology and Evolutionary Biology Yale University New Haven CT USA; ^2^ Scientific Services Kruger National Park Skukuza South Africa; ^3^ Present address: Department of Ecology and Evolutionary Biology Princeton University Princeton NJ USA; ^4^ Present address: Department of Mechanical Engineering University of Minnesota Minneapolis MN USA

**Keywords:** elephant impacts, landscape heterogeneity, *Loxodonta Africana*, management legacies, savanna vegetation, surface water, tree damage

## Abstract

1. Though elephants are a major cause of savanna tree mortality and threaten vulnerable tree species, managing their impact remains difficult, in part because relatively little is known about how elephant impacts are distributed throughout space.
2. This is exacerbated by uncertainty about what determines the distribution of elephants themselves, as well as whether the distribution of elephants is even informative for understanding the distribution of their impacts.
3. To better understand the factors that underlie elephant impacts, we modeled elephant distributions and their damage to trees with respect to soil properties, water availability, and vegetation in Kruger National Park, South Africa, using structural equation modeling.
4. We found that bull elephants and mixed herds differed markedly in their distributions, with bull elephants concentrating in sparsely treed basaltic sites close to artificial waterholes and mixed herds aggregating around permanent rivers, particularly in areas with little grass.
5. Surprisingly, we also found that the distribution of elephant impacts, while highly heterogeneous, was largely unrelated to the distribution of elephants themselves, with damage concentrated instead in densely treed areas and particularly on basaltic soils.
6. Results underscore the importance of surface water for elephants but suggest that elephant water dependence operates together with other landscape factors, particularly vegetation community composition and historical management interventions, to influence elephant distributions.

## Graphical abstract

Elephant conservation must strike the balance between protecting elephants and managing the impacts they have on savanna ecosystems, especially on trees, but we still know relatively little about how elephant impacts are distributed in space. In this study, we found that elephant impacts were largely unrelated to the distribution of elephants themselves and were instead concentrated in densely treed areas and particularly on basaltic soils. As such, we need to shift our focus away from describing patterns of elephant landscape use and instead need to consider characteristics of trees themselves and their role in tolerating elephant damage.

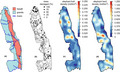

## 1 INTRODUCTION

Elephant conservation aims to balance the protection of elephants with the management of the impacts they have on their surroundings. Although African savanna elephants (*Loxodonta africana*) are considered a vulnerable species overall (Chase et al., 2016), their populations have grown rapidly in some parks (Smit & Ferreira, 2010), so much so that they often have the greatest per‐species biomass in an area (Hempson et al., 2015). Combined with their lack of predators, which allows them to forage more intensely than smaller herbivores (Abraham et al., 2019; Owen‐Smith et al., 2006), feeding by elephants can result in severe impacts on savanna vegetation, particularly trees (Morrison et al., 2016; Vanak et al., 2012).

Altogether, elephants pose a central concern as agents of savanna tree mortality (Asner & Levick, 2012; Jacobs & Biggs, 2002; Morrison et al., 2016), both via direct damage and in interaction with other stressors such as fire and insects (Vanak et al., 2012; Wigley et al., 2019). Elephants impact trees directly by stripping bark, breaking stems and branches, or simply uprooting trees entirely, all of which can make trees more susceptible to insect damage and fire‐induced mortality (Jacobs & Biggs, 2002; Moncrieff et al., 2017; Wigley et al., 2019). Some tree species are at particular risk (Duffy et al., 2002; Edkins et al., 2008; Midgley et al., 2020; Shannon et al., 2008), including *Sclerocarya birrea* (marula), *Adansonia digitata* (baobab), and *Acacia nigrescens* (alt. *Senegalia nigrescens*; knobthorn), which are considered valuable ecologically and function as iconic species with touristic value (Owen‐Smith et al., 2006).

However, debate continues as to the real threat posed by elephants to trees and biodiversity overall in savannas (Coverdale et al., 2016; Guldemond et al., 2017; Henley & Cook, 2019). This debate is exacerbated by general uncertainty about what underlies heterogeneity both in elephant density and in how their impacts are distributed in savanna landscapes, which can vary dramatically in space (Anderson & Walker, 1974; Codron et al., 2006). Many studies have attempted to identify consistent patterns of impact (Anderson & Walker, 1974; Asner et al., 2016; Davies & Asner, 2019; Duffy et al., 2002; Van Wyk & Fairall, 1969), but results are so often uncertain or contradictory that meta‐analysis has concluded there is no universal predictor that generalizes well across study areas (Guldemond et al., 2017).

One exception to this uncertainty is a well‐documented association of elephants with water (Harris et al., 2008; Hempson et al., 2015). Elephants are widely considered to be a “water‐dependent” species (Chamaillé‐Jammes et al., 2007), and water availability is believed to be a key driver structuring elephant landscape use (Smit & Ferreira, 2010; De Beer & Van Aarde, 2008). Indeed, most elephant populations are observed to cluster around rivers (Harris et al., 2008; Smit & Ferreira, 2010; Smit et al., 2007) and to a lesser extent around artificial waterholes (Chamaillé‐Jammes et al., 2007; Loarie et al., 2009), which is thought to increase encounter rates between elephants and vulnerable tree species (O’Connor et al., 2007). However, the factors that shape elephant distributions are more complex than this wholesale focus on water availability suggests. Water availability may be only a strong determinant of elephant distributions locally (de Knegt et al., 2011; Smit & Ferreira, 2010), or water availability may determine elephant landscape use at multiple scales (Chamaillé‐Jammes et al., 2007; Hempson et al., 2015). Furthermore, elephant landscape use has been shown to vary with temperature (Williams et al., 2018), short‐term changes in NDVI (Bohrer et al., 2014), and particularly tree density and identity (Harris et al., 2008; Owen‐Smith et al., 2006). These drivers of elephant landscape use may, like water availability, also depend on scale, with different factors dominating local versus regional elephant distributions (Marshal et al., 2011; Shrader et al., 2012). How all these processes integrate to determine elephant distributions is not known, making it difficult to predict or manage their impacts.

Furthermore, it is often assumed that the distributions of elephant impacts and elephants themselves are analogous and interchangeable (Chamaillé‐Jammes et al., 2007; Loarie et al., 2009; Smit & Ferreira, 2010). However, elephants do not only damage trees for food (Coverdale et al., 2016; Midgley et al., 2005); elephants —young bulls in particular— often knock down trees as a sexual display, to impress females or intimidate other males (Midgley et al., 2005). Likewise, elephants have been hypothesized to purposefully knock down nonfeeding trees to modify the structure of vegetative communities (“elephant farming” hypothesis; Midgley et al., 2005). As such, even places where elephants are not foraging intensely may still suffer severe impacts. Indeed, the assumption that elephant impacts are most severe where elephants are most densely concentrated has limited empirical support (Asner et al., 2016; Davies & Asner, 2019), in part because (a) spatially explicit data on both elephants and their impacts across sufficiently large scales are challenging to collect; and (b) few places have stable enough elephant populations and management histories to reasonably draw such a comparison (Bradshaw et al., 2005; Chase et al., 2016; Guldemond et al., 2017; Smit & Ferreira, 2010). Altogether, it remains unclear whether the distribution of elephants informs the distribution of their impacts.

Here, we address these uncertainties by investigating the distribution of elephants and their impacts on trees in Kruger National Park, South Africa (Kruger). We modeled potential predictors of elephant landscape use and elephant impacts using structural equation modeling (SEM) and identified environmental and ecological factors that most contributed to explaining the distributions of elephants and their impacts. Because these models can be susceptible to spatial autocorrelation, we also used a more limited spatially explicit structural equation modeling (SE‐SEM) framework to verify nonspatial SEM results. By doing so, we aimed to clarify the drivers of elephant impacts in order to better inform current management practices.

## 2 MATERIALS AND METHODS

### 2.1 Study site

**FIGURE 1:**
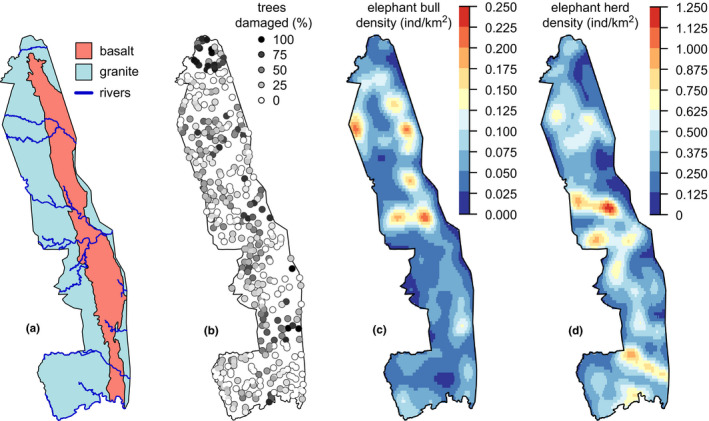
The distributions of (a) landscape features (soil parent geology and permanent rivers), (b) elephant impacts at VCA sites, (c) bull elephant densities, and (d) densities of elephants in mixed herds in Kruger National Park, South Africa. Note that the scales differ in (c) and (d), as elephants in mixed herds are nearly an order of magnitude more numerous than bull elephants

Kruger National Park encompasses ~20,000 km^2^ (22°20′ to 25°30′S; 31°10′ to 32°00′ E) of low‐lying areas (260–839 m) of northeastern South Africa. It is dominated by two underlying parent materials, a granite and a basalt, broadly characterized as nutrient‐poor and nutrient‐rich, respectively (Staver et al., 2017) (Figure 1a). Across the park, mean annual rainfall ranges from 450 mm in the north to 750 mm in the south, with significant interannual variation in rainfall (Staver et al., 2019). The park is bisected by six major river systems flowing west to east, down from the Drakensberg escarpment toward the Mozambican coastal plain (Figure 1a). This variation in both water and nutrient availability contributes to the wide variety of vegetation found throughout the park (Venter, 1990). Park management has also historically provided water at artificial water points (Pienaar et al., 1997; Redfern et al., 2005), and many small streams (associated with major river networks) are seasonal, resulting in temporal variability in water availability; however, water in the larger rivers remains accessible, even in extreme drought (Smit & Ferreira, 2010).

### 2.2 Data sources

**FIGURE 2:**
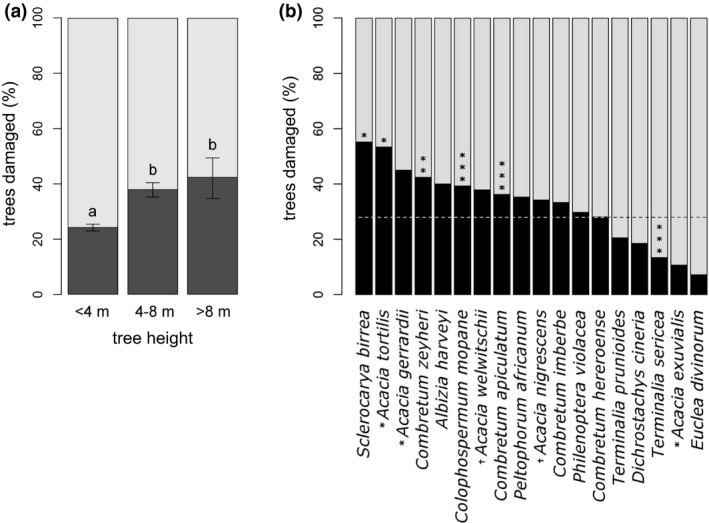
Elephant damage rates on trees by (a) height class and (b) by species. In (a), error bars denote 95% confidence intervals around each sample mean, and letters indicate significant difference (*p* < .001). In (b), the mean percentage of trees damaged (28.0%) is given by the dotted line. Species means significantly different from the universal mean are indicated by stars (*p ≤ .05, **p ≤ .01, and ***p ≤ .001). Discussion continues on the proper classification of the polyphyletic *Acacia* genus; here all species have been identified as *Acacia*, but those proposed to belong in *Vachellia* are marked with an asterisk (*) and those in *Senegalia* with a dagger (†)

Spatially explicit data on elephant impacts were collected in 2008 at Kruger's Veld Condition Assessment (VCA) sites (Figure 1b). These 50 m × 60 m plots (*n* = 533) are distributed throughout the entirety of the park and capture Kruger's major vegetation types; they have been monitored since 1989 for variations in grass biomass to inform fire management, but tree layer plots were added in 2008 (see Staver et al., 2017, Staver, 2018, and Staver et al., 2019 for formal analyses of vegetation structure and a more complete description of the full data). At each VCA site, all trees >3 m in height were identified and measured within 8 subplots of 5 m radius (for a total sampling area ~628 m^2^ per site). For this analysis, trees were grouped into three height classes: trees <4 m in height, trees between 4 and 8 m, and trees >8 m (see Figure 2b). In addition, the severity of elephant damage was scored on each of these trees; in order to calculate per‐site rates of damage for this study (Figure 1b) and also due to concerns around the consistency of severity scores, we have reduced these scores to a binary presence/absence of elephant damage.

To evaluate how tree species composition impacted elephant distributions, we computed the density of tree species damaged by elephants at each VCA site (“preferred tree species”). We weighted the stem density for each tree species at a given VCA site by that species’ whole‐park mean damage rate. This resulted in a comparable metric corresponding to the relative density of elephant‐preferred trees at each VCA site, generating a preference score wherein larger values correspond to a higher density of more preferred tree species.

For each VCA site, we also extracted information on underlying parent geologic material and mean annual rainfall from maps maintained by the park. Rainfall maps are based on data collected at 22 weather stations that have been continuously monitored since 1989 throughout Kruger (see Staver et al., 2017 for more information). We also calculated the minimum distance from each VCA site both to the nearest permanent rivers (order ≥ 6) and to the nearest artificial waterholes open in 2011 (the distribution of which is largely representative of the waterholes open during the end of the study period; Pienaar et al., 1997; Redfern et al., 2005).

Finally, elephant densities at the VCA sites were estimated based on megaherbivore helicopter aerial censuses performed by park management each year from 1985 through 2008. Aerial censuses were performed during the late dry season (August) when elephant impacts on trees are thought to be most severe (Davies & Asner, 2019; Smit & Ferreira, 2010) due to their increased utilization of trees for forage (Codron et al., 2006). Transects were flown across the park following drainage lines, with four expert observers noting the position, number of individuals, and composition of all elephant groups (see Smit et al., 2007 for further details). To account for movement, parkwide density layers were generated from point counts for each year from 1985 to 2008; separate layers were generated for bulls and for mixed herds (comprising females, young males, and calves) (Figure 1c and d). Point‐based censuses were interpolated using a 5 km Gaussian kernel smoother with the function *density.ppp* in the “spatstat” package (Baddeley & Turner, 2004) to generate maps of elephant density across the park for each census year (per Davies & Asner, 2019; MacFadyen et al., 2019). Parkwide density layers from 1985 to 2008 were aggregated across years to yield average distribution estimates for bulls and mixed herds (Figure 1c and d). Because elephant populations have increased in Kruger through time (Smit et al., 2007), this approach inherently ascribes more weight to more recent censuses. However, we feel this approach is appropriate since damage data are from 2008 and more contemporaneous elephant distributions likely do matter more for understanding their impacts. Densities at the VCA sites were then extracted from aggregated density maps to correspond to site‐level data, at which level we performed analyses.

### 2.3 Data analysis

Data were analyzed in R 3.2.3. To evaluate the possibility that elephant preferences for certain vegetation types might contribute to how their impacts are distributed in space, we compared the proportion of trees damaged by elephants across all tree species and in each of the three tree height classes using a series of *χ* ^2^ and pairwise binomial tests (see Figure 2). Confidence intervals were calculated at 95% around mean damage rates for the three height classes and for each species via bootstrapping within each category using the “boot” package in R (Canty & Ripley, 2017). Then, the mean percentages of trees damaged by elephants for each species and height class were compared to the overall mean (27.97% across all trees) using binomial tests to determine whether damage rates differed significantly from the mean. Species that were damaged significantly more than the global mean were considered preferred species (Figure 2b).

To examine relationships of the site‐level variables described above on elephant densities and their impacts, we used structural equation models (*SEM*) in the “lavaan” R package (Rosseel, 2012). We constructed alternative candidate models to test the inclusion of variables locally significant for *p* < .1, and locally insignificant relationships were tolerated if they improved overall model fit. Missing data were handled using full information maximum likelihood (FIML), which performs well in SEM (Enders & Bandalos, 2001). The model with the lowest AIC was considered the best model, and we compared it with spatially explicit SEM (SE‐SEM) models (using R package “sesem”; Lamb et al., 2014) to test the importance of spatial autocorrelation. In SE‐SEM, SEMs are fit with varying lagged distances to evaluate whether the strengths of relationships change as the spatial scale of dependencies changes. By synthesizing the results from both SEM and SE‐SEM, we were able to identify the strongest predictors of both elephant distributions and the distributions of their impacts throughout Kruger.

## 3 RESULTS

Spatially explicit structural equation modeling showed that spatial models were preferred (Figure S3), but most relevant relationships remained consistent between spatial and nonspatial models (Figure S4). As such, unless otherwise noted, all links mentioned hereafter are consistent across spatial and nonspatial models. Overall model fit was within acceptable limits (CFI = 0.97, RMSEA = 0.06 [0.03, 0.08], SRMR = 0.03) despite a substandard *χ^2^* (*χ^2^* = 36.7, *df* = 16, *p* = .002; large *p*‐values are preferred in *SEM*). This disparity results from the inclusion of the “preferred trees” metric, which was derived from the same data as the “tree density” and “elephant impact” variables and therefore could not be linked to either of them without violating assumptions of independence. Such links, however, were strongly suggested by modification indices, and excluding them therefore made the final model appear “worse” in contrast to the hypothetical, statistically invalid model that did include these links.

### 3.1 Landscape features

The relationships observed among vegetative and abiotic factors were unsurprising. More grass was found in rainy, basaltic areas far from rivers, especially where trees were sparse (Table S1; Figure S2), confirming prior work indicating that both rainfall and nutrient‐rich soils favor grass productivity (which in turn suppresses tree recruitment; Staver et al., 2017) and that grass biomass is low around rivers due to high levels of trampling and grazing from high herbivore traffic (Jacobs & Naiman, 2008; Van Coller et al., 2013; Van Wyk & Fairall, 1969). Sites in rainier parts of Kruger were nearer permanent rivers in nonspatial models (Figure S2), but intuitively this relationship fell away in spatially explicit modeling (Figure S4).

Tree density seemed to be dependent on rainfall, with higher densities in dry areas, but the effect was weak in spatial modeling (Figure S4); instead, geology was the best predictor of tree density, with more trees at granitic sites (Figure S2). This again agrees with past work suggesting that soil plays a key role in determining savanna vegetation structure both in Kruger (Staver et al., 2017) as well as at continental scales (Case & Staver, 2018). The importance of geology and rainfall was reversed when considering the density of elephant‐preferred trees in particular: Their density was most strongly influenced by rainfall and only weakly by geology (Figure S2). Tree species preferred by elephants therefore are more common at drier sites but are only marginally more common on granitic soils. The model also suggested that river proximity may influence the distribution of these tree species, but the link proved insignificant (Figure S4).

### 3.2 Elephant impacts

Elephants had significant impacts on trees, damaging large trees (>8 m) (*χ^2^* = 27.95, *df* = 1, *p* < .0001) and medium trees (4–8 m) (*χ^2^* = 99.9, *df* = 1, *p* < .0001) more than short ones (< 4 m) (Figure 2a), although there was no difference in the rates of damage between tall and medium trees (*χ^2^* = 1.08, *df* = 1, *p* = .30). Elephants also impacted some tree species more than others (Figure 2b). Of the 18 species for which at least 15 individuals were sampled, four species were damaged significantly more often than the average and five species were damaged significantly less often (see Figure 2b for significance levels by species and Table S3 for estimated rates). Most notably, elephants damaged significantly more *Sclerocarya birrea* (marula) and *Acacia tortilis* (alt. *Vachellia tortilis*, umbrella thorn), while damaging low proportions of bush‐encroaching species, including *Dichrostachys cinerea*, *Terminalia sericea*, and *Euclea divinorum* (though note that damage may not perfectly correlate with how widely utilized a species is, due to species‐specific differences in growth rates and tolerance to herbivory; Owen‐Smith et al., 2006; Staver et al., 2012; Wakeling et al., 2011).

Several weak predictors of elephant damage across Kruger were observed, such as bull elephant density, which was linked to damage in the best nonspatial model (Table S1) but proved an insignificant predictor not only in *SEM* (Figure S2), but also in SE‐SEM (Figure S4) and linear modeling (*F*_1,387_ = 0.527, *R* ^2^ = 0.00, *p* = .47). Grass biomass also seemed to increase tree damage (Figure S2), but the effect again vanished in spatial models (Figure S4).

**FIGURE 3:**
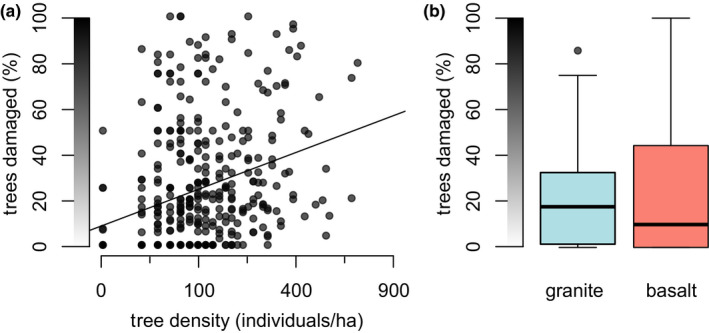
Relationships between elephant impact and (a) tree density and (b) geology. Elephants damage significantly more trees in densely treed areas and on basalt‐derived soils. Relationships are significant in SEM (*p* ≤ .001), though the relationship between impact and geology is only weakly supported in SE‐SEM. Axis colors correspond to color gradients in Figure 1b

Tree density, on the other hand, was the strongest predictor of elephant damage in both spatial (Figure S4) and nonspatial models (Figure S2), with elephants damaging the highest proportion of trees in the most densely treed areas (*F*_1,381_ = 42.15, *R* ^2^ = 0.10, *p* < .0001) (Figure 3a). This pattern interacted with geology: Despite lower tree densities on basaltic sites (Figure S2), we observed little difference in tree damage between basalt and granite (*t* = −0.83, *df* = 142.92, *p* = .41) (Figure 3b). This suggests that the influence of tree density on damage rates was offset by some other effect (Figure S2), perhaps indicating that nutrient‐rich basalts make trees more palatable. This result aligns with SEM results, which indicated that impact was somewhat more frequent on basaltic sites (Figure S2), though the link was only weakly supported by SE‐SEM (Figure S4).

### 3.3 Elephant densities

**FIGURE 4:**
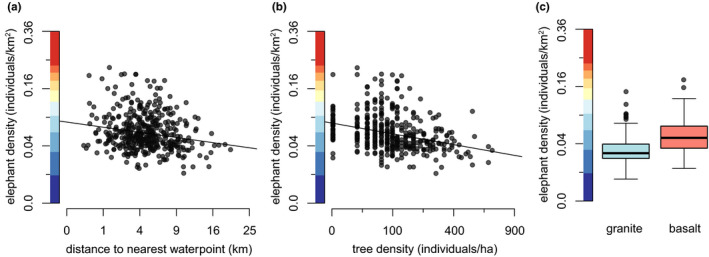
Relationships between bull elephant densities and (a) distance to the nearest waterhole, (b) tree density, and (c) geology. Bull elephants aggregate in sparsely treed basaltic regions of Kruger, particularly around waterholes. All relationships are significant in SEM and SE‐SEM (*p* ≤ .001). Axis colors correspond to color gradients in Figure 1c

**FIGURE 5:**
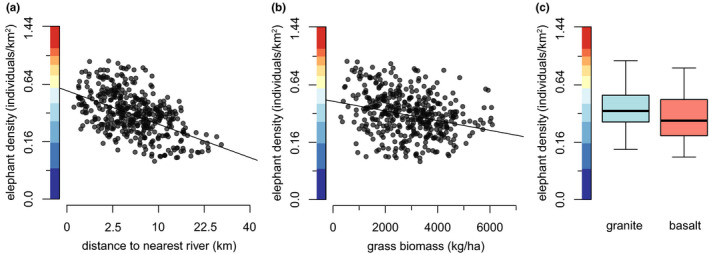
Relationships between densities of elephants in mixed herds and (a) distance to the nearest river, (b) grass biomass, and (c) geology. Mixed herds congregate strongly around rivers, particularly in granitic sites with low grass biomass. All relationships are significant in SEM and SE‐SEM (*p* ≤ .001). Axis colors correspond to color gradients in Figure 1d

Elephant density was influenced by a variety of water and vegetative characteristics, but the determinants of distributions differed markedly between bull elephants and elephants in mixed herds (Figures 4 and 5). Bull elephant density was most strongly associated with areas of low tree density (*F*_1,395_ = 43.47, *R* ^2^ = 0.10, *p* < .0001) and was also somewhat greater on basalts (*t* = −7.07, *df* = 200.68, *p* < .0001; granite mean 2.23 elephants/km^2^, basalt mean 2.71 elephants/km^2^) and marginally greater in areas close to artificial water points (*F*_1,413_ = 15.80, *R* ^2^ = 0.03, *p* < .0001) (Figure 4). It also seemed that they might be concentrated in areas of the park with less rain, though this link fell away in spatial models (Figure S4).

In contrast, the density of elephants in herds was associated with an entirely different set of factors. Herds tended very strongly to areas close to rivers (*F*_1,413_ = 141.20, *R* ^2^ = 0.253, *p* < .0001), particularly those with less grass (*F*_1,413_ = 34.20, *R* ^2^ = 0.07, *p* < .0001) (Figure 5), agreeing with the well‐known water dependence of herds that include calves (Barnes, 1983; Harris et al., 2008). An apparent tendency toward lightly treed granitic areas was insignificant in spatial modeling (Figure S4); there was also a slight relationship between herd density and areas that specifically had higher densities of “preferred” trees which was supported in spatial modeling (Figure S4) but insignificant in linear regression (*F*_1,400_ = 0.799, *R* ^2^ = 0.00, *p* = .37).

## 4 DISCUSSION

Here, we examined the distributions of elephants and their impacts in Kruger National Park, South Africa. We found that (1) elephants have distinct preferences for certain tree size classes and species, particularly *Sclerocarya birrea* (marula), *Acacia tortilis* (alt. *Vachellia tortilis*, umbrella thorn), and *Colophospermum mopane* (mopane). Surprisingly, however, we found that (2) the distribution of preferred trees did not seem to influence the distribution of elephants: The density of preferred trees was not associated with bull elephant densities and was only weakly associated with the density of elephants in mixed herd. Instead, we found that (3) bull elephants concentrate in sparsely treed basaltic sites close to artificial waterholes, (4) whereas mixed herds aggregate strongly around permanent rivers, particularly in areas with little grass. Finally, we found that (5) the distribution of elephant impacts was largely unrelated to the distribution of elephants themselves, concentrated instead in densely treed areas and on basaltic soils.

First and foremost, these findings corroborate existing work suggesting that elephants are dependent on surface water and that their landscape use is tightly associated with water as a result (Harris et al., 2008; Smit & Ferreira, 2010; De Beer & Van Aarde, 2008): Here, elephants in mixed herds concentrated near rivers (Figure 4a), whereas bull elephants ranged close to waterholes (Figure 3a). This aligns with the fact that Kruger's elephants generally seek drinking water every two days (De Beer & Van Aarde, 2008), which forces them to remain within easy traveling distance of surface water sources (Barnes, 1983). While bulls may wander more freely from water while traveling alone or in small bachelor groups (De Beer & Van Aarde, 2008), relatively more numerous herds that include calves and lactating cows are more water‐dependent (De Beer & Van Aarde, 2008), such that the majority of elephants concentrate around rivers (though vegetation may also play a role in driving the preference of mixed herds for rivers, see below).

However, contrastingly, we found that water availability did not determine elephant densities at regional scales. The density of elephants in mixed herds was largely unrelated to rainfall, and bulls were possibly negatively associated with rainfall (Figures S2 and S4). This pattern contrasts both with the local water dependence discussed above and with broader patterns at continental scales, which show that elephant biomass increases with increasing rainfall across all of Africa (Hempson et al., 2015). These discrepancies suggest that, while water access is important for elephants, this need is largely satisfied by access to surface water sources (Loarie et al., 2009), such that wholesale water availability may not be a major determinant of overall variation in elephant densities in the landscape (Chamaillé‐Jammes et al., 2007; de Knegt et al., 2011).

Alternatively, the discrepancy between local and regional relationships could mean that additional factors outweigh elephants’ water dependence in determining overall patterns of elephant landscape use. In particular, past management interventions (*e.g*., culling, large‐scale elephant exclosures, and water provisioning; Bradshaw et al., 2005; Graham et al., 2009; Smit et al., 2020) and vegetation composition (de Knegt et al., 2011) may play a prominent role in structuring elephant distributions overall, mediated by locally heterogeneous population growth rates (Chamaillé‐Jammes et al., 2007; Robson & van Aarde, 2018) and elephant foraging behaviors and preferences (Shrader et al., 2012).

Indeed, the relationship between bull elephants and water points suggests a role for management in structuring current elephant distributions, indicating that water provisioning may have modified the relationship between Kruger's elephants and rainfall; water points may have allowed elephants to make use of drier portions of the landscape that they could not previously access (Loarie et al., 2009; Smit & Ferreira, 2010). This is particularly noteworthy since we found here that the tree species preferred by elephants are concentrated in the drier portions of Kruger (Figure S2). Historic water provisioning may therefore have enabled elephants to access this preferred vegetation, potentially explaining the high damage rates observed therein.

These findings raise the question of what role management has had in determining elephant distributions in Kruger. Kruger has been subject to a wide range of management interventions, including culling elephants to suppress populations, fencing off regions to protect against elephant impacts, and provisioning surface water to disperse elephants (Redfern et al., 2005; Robson & van Aarde, 2018; Smit & Ferreira, 2010; Smit et al., 2020). All of these initiatives are thought to have profound effects on how elephants use space (Chamaillé‐Jammes et al., 2007; Loarie et al., 2009; Robson & van Aarde, 2018; Smit & Ferreira, 2010; Smit et al., 2020), such that current distributions may still reflect the legacies of these interventions and therefore cannot be understood without explicit consideration of past management efforts.

Additionally, our results suggest that characteristics of the vegetative community may play a role in determining elephant space use. Both bulls and herds seem to preferentially use nutrient‐rich subhabitats: Bulls preferentially utilize the nutrient‐rich basaltic sites (Figure 3c), where vegetation is possibly more palatable. Similarly, herds utilize riverside sites (Figure 4a), which are likewise thought to be comparatively nutrient‐rich and also feature distinctive riparian vegetation (Owen‐Smith et al., 2006; Van Wyk & Fairall, 1969). This apparent preference by both bull elephants and mixed herds for relatively nutrient‐rich vegetation is somewhat surprising in light of metabolic theory suggesting that large‐bodied herbivores like elephants should be more concerned with forage quantity rather than quality (Damuth, 1987; Olff et al., 2002), as well as evidence that comparatively smaller female elephants prefer high‐quality forage compared to larger male elephants, which seek out large quantities of forage (Greyling, 2004; Shannon et al., 2006). In further support of the role of vegetation in driving elephant landscape use, we found that herds seem to utilize sites with greater numbers of “preferred” trees (Figure S2), again suggesting that vegetation characteristics may contribute to their landscape use preferences, though the link was weak (and absent for bulls). In sum, our results suggest that vegetation may play a role in determining elephant distributions, through both nutrient content and species composition. Both these possibilities bear further examination.

Much like the distribution of elephants themselves, we found substantial variation in elephant damage, both compositionally and spatially: Damage to trees differed dramatically by species and size class, with elephants preferentially damaging larger trees and certain tree species while avoiding other species (Figure 2; Table S3); furthermore, impacts differed from plot to plot, ranging from local tree damage rates of near‐zero to almost 100% (Figure 5). However, despite substantial heterogeneity in impacts, the variation was largely unexplainable from the predictors evaluated here; the only strong correlate of elephant damage to trees was tree density (Figure 5a), possibly due to increased browsing efficiency in densely treed areas (a type 3 functional response). Surprisingly, local elephant densities—bulls and herds both—had little explanatory power for understanding impact, at least at this coarse binary resolution (Figures S2 and S4). While it is often assumed that the places where elephants are congregated are subject to the most severe impacts (Chamaillé‐Jammes et al., 2007; Loarie et al., 2009; Smit & Ferreira, 2010), our results call into question this assumption. It remains unclear from our analysis what decouples elephant distributions from the distribution of their impacts; spatial variation in tree tolerance to elephant damage may play a role, as may variability in tree growth rates, confounding underlying rates of elephant utilization (Owen‐Smith et al., 2006; Staver et al., 2012; Wakeling et al., 2011).

Altogether, our results suggest that, while elephant impacts are indeed heterogeneous, they are not heterogeneous in a way that maps easily onto environmental variation. This indicates a need to rethink future investigations of elephant damage to trees.

## 5 CONCLUSIONS

There are two major takeaways from this study. Firstly, this research adds to the substantial body of literature demonstrating the importance of water for elephants (Barnes, 1983; Chamaillé‐Jammes et al., 2007; Hempson et al., 2015; Loarie et al., 2009; Smit & Ferreira, 2010; De Beer & Van Aarde, 2008). However, we find that it is surface water in particular, rather than regional water availability, to which elephants respond (Chamaillé‐Jammes et al., 2007; Smit & Ferreira, 2010; De Beer & Van Aarde, 2008). That said, we speculate that both past management interventions and vegetation characteristics may contribute to this pattern; water provisioning may have enabled bull elephants in particular to access regions of the park—and the vegetation therein—previously inaccessible to them (Loarie et al., 2009; Smit & Ferreira, 2010). Similarly, preferences for specific tree species and vegetation of a particular quality may contribute to decoupling elephants from rainfall (Midgley et al., 2005; Shannon et al., 2008; Shrader et al., 2012). Both possibilities warrant direct examination.

Our results also emphasize the importance of separately considering the landscape use of bulls and mixed herds (Midgley et al., 2005; Smit et al., 2007). We find that their distributions are entirely determined by different factors: Mixed herds are constrained to rivers by the poor mobility of calves, whereas more mobile bulls are comparatively free to roam farther from rivers (Barnes, 1983; De Beer & Van Aarde, 2008). As such, future management efforts should account for the differential mobility of bulls and herds.

Secondly, we find that the distribution of elephant damage is largely inexplicable by any of the factors we analyze here, even by the distribution of elephants themselves. This suggests that there is no obvious silver bullet for why elephants are where they are and why their preferences are what they are, consistent with a large body of literature (Anderson & Walker, 1974; Asner et al., 2016; Davies & Asner, 2019; Duffy et al., 2002; Guldemond et al., 2017; Van Wyk & Fairall, 1969). This does present an intriguing question, however: What decouples elephants from their impacts?

One possibility is that we may be looking at elephant distributions at the wrong time. It has historically been assumed that elephant impacts on vegetation are most severe during the dry season, due to increased utilization of trees for forage (Codron et al., 2006). However, certain preferred tree species are consistently utilized by elephants for forage year‐round (Codron et al., 2006; Jacobs & Biggs, 2002). Also, elephant damage to trees is not just for food (Coverdale et al., 2016; Midgley et al., 2005), such that we may need to consider how elephants damage trees even when they are not foraging on them intensely. Alternatively, the distribution of elephant damage may be determined by particularly severe resource bottlenecks (*e.g*., during drought, when resources are especially limited, and elephants change their landscape use accordingly to access forage reserves; Abraham et al., 2019), such that considering elephant distributions during average conditions may likewise be inadequate.

Furthermore, focusing on the elephants alone may be inappropriate for understanding their impacts. Instead, we may need to shift our focus to incorporate plants too. Savanna trees differ markedly in their growth rates and tolerance to herbivory (Midgley et al., 2020; Owen‐Smith et al., 2006; Staver et al., 2012; Wakeling et al., 2011), and as such, spatial variation in the ability of trees to tolerate and recover from elephant damage may obscure any relationship between where elephants are and where damage is observed (Fornoni et al., 2004; Więski & Pennings, 2014). Trees in areas densely populated by elephants may be locally adapted to avoiding or recovering from elephant damage, for example. Evaluating variation in plants themselves may therefore be crucial to understanding the impacts elephants have on them. Altogether, predicting elephant distributions and that of their impacts remains an ongoing challenge.

## CONFLICT OF INTEREST

None declared.

## AUTHOR CONTRIBUTIONS

**Joel O. Abraham:** Data curation (supporting); Formal analysis (supporting); Investigation (equal); Project administration (lead); Supervision (lead); Visualization (lead); Writing‐review & editing (lead). **Emily R. Goldberg:** Conceptualization (lead); Formal analysis (lead); Investigation (lead); Methodology (lead); Writing‐original draft (lead); Writing‐review & editing (supporting). **Judith Botha:** Data curation (lead); Writing‐review & editing (supporting). **A. Carla Staver:** Conceptualization (lead); Methodology (lead); Project administration (supporting); Resources (lead); Supervision (lead); Writing‐original draft (equal); Writing‐review & editing (lead).

## Supporting information

Supplementary Material

## Data Availability

Data are archived in the public data repository maintained by the South African National Park Services (http://dataknp.sanparks.org/sanparks/metacat/judithk.112343.1/sanparks).

## References

[ece37465-bib-0001] Abraham, J. O. , Hempson, G. P. , & Staver, A. C. (2019). Drought‐response strategies of savanna herbivores. Ecology and Evolution, 9(12), 7047–7056. 10.1002/ece3.5270 31380032 PMC6662422

[ece37465-bib-0002] Anderson, G. D. , & Walker, B. H. (1974). Vegetation composition and elephant damage in the Sengwa Wildlife Research Area, Rhodesia. Journal of the Southern African Wildlife Management Association, 4(1), 1–14.

[ece37465-bib-0003] Asner, G. P. , & Levick, S. R. (2012). Landscape‐scale effects of herbivores on treefall in African savannas (ed E Cleland). Ecology Letters, 15, 1211–1217. 10.1111/j.1461-0248.2012.01842.x 22863324

[ece37465-bib-0004] Asner, G. P. , Vaughn, N. , Smit, I. P. , & Levick, S. (2016). Ecosystem‐scale effects of megafauna in African savannas. Ecography, 39(2), 240–252. 10.1111/ecog.01640

[ece37465-bib-0005] Baddeley, A. J. , & Turner, R. (2004). spatstat: An R package for analyzing spatial point pattens.

[ece37465-bib-0006] Barnes, R. F. W. (1983). Elephant behaviour in a semi‐arid environment. African Journal of Ecology, 21(3), 185–196. 10.1111/j.1365-2028.1983.tb01180.x

[ece37465-bib-0007] Bohrer, G. , Beck, P. S. , Ngene, S. M. , Skidmore, A. K. , & Douglas‐Hamilton, I. (2014). Elephant movement closely tracks precipitation‐driven vegetation dynamics in a Kenyan forest‐savanna landscape. Movement Ecology, 2(1), 1–12. 10.1186/2051-3933-2-2 25520813 PMC4267703

[ece37465-bib-0008] Bradshaw, G. A. , Schore, A. N. , Brown, J. L. , Poole, J. H. , & Moss, C. J. (2005). Elephant breakdown. Nature, 433(7028), 807. 10.1038/433807a 15729320

[ece37465-bib-0009] Canty, A. , & Ripley, B. (2017). boot: Bootstrap R (S‐Plus) Functions. R package version 1.3‐20.

[ece37465-bib-0010] Case, M. F. , & Staver, A. C. (2018). Soil texture mediates tree responses to rainfall intensity in African savannas. New Phytologist, 219(4), 1363–1372. 10.1111/nph.15254 29862513

[ece37465-bib-0011] Chamaillé‐Jammes, S. , Valeix, M. , & Fritz, H. (2007). Managing heterogeneity in elephant distribution: Interactions between elephant population density and surface‐water availability. Journal of Applied Ecology, 44, 625–633. 10.1111/j.1365-2664.2007.01300.x

[ece37465-bib-0012] Chase, M. J. , Schlossberg, S. , Griffin, C. R. , Bouché, P. J. C. , Djene, S. W. , Elkan, P. W. , Ferreira, S. , Grossman, F. , Kohi, E. M. , Landen, K. , Omondi, P. , Peltier, A. , Selier, S. A. J. , & Sutcliffe, R. (2016). Continent‐wide survey reveals massive decline in African savannah elephants. PeerJ, 4, e2354. 10.7717/peerj.2354 27635327 PMC5012305

[ece37465-bib-0013] Codron, J. , Lee‐Thorp, J. A. , Sponheimer, M. , Codron, D. , Grant, R. C. , & de Ruiter, D. J. (2006). Elephant (*Loxodonta africana*) diets in Kruger National Park, South Africa: Spatial and landscape differences. Journal of Mammalogy, 87, 27–34. 10.1644/05-MAMM-A-017R1.1

[ece37465-bib-0014] Coverdale, T. C. , Kartzinel, T. R. , Grabowski, K. L. , Shriver, R. K. , Hassan, A. A. , Goheen, J. R. , Palmer, T. M. , & Pringle, R. M. (2016). Elephants in the understory: Opposing direct and indirect effects of consumption and ecosystem engineering by megaherbivores. Ecology, 97(11), 3219–3230. 10.1002/ecy.1557 27870025

[ece37465-bib-0015] Damuth, J. (1987). Interspecific allometry of population density in mammals and other animals: The independence of body mass and population energy‐use. Biological Journal of the Linnean Society, 31(3), 193–246. 10.1111/j.1095-8312.1987.tb01990.x

[ece37465-bib-0016] Davies, A. B. , & Asner, G. P. (2019). Elephants limit aboveground carbon gains in African savannas. Global Change Biology, 25(4), 1368–1382. 10.1111/gcb.14585 30723962

[ece37465-bib-0017] De Beer, Y. , & Van Aarde, R. J. (2008). Do landscape heterogeneity and water distribution explain aspects of elephant home range in southern Africa's arid savannas? Journal of Arid Environments, 72(11), 2017–2025.

[ece37465-bib-0018] de Knegt, H. J. , van Langevelde, F. , Skidmore, A. K. , Delsink, A. , Slotow, R. , Henley, S. , Bucini, G. , de Boer, W. F. , Coughenour, M. B. , Grant, C. C. , Heitkönig, I. M. A. , Henley, M. , Knox, N. M. , Kohi, E. M. , Mwakiwa, E. , Page, B. R. , Peel, M. , Pretorius, Y. , van Wieren, S. E. , & Prins, H. H. T. (2011). The spatial scaling of habitat selection by African elephants. Journal of Animal Ecology, 80, 270–281. 10.1111/j.1365-2656.2010.01764.x 21054380

[ece37465-bib-0019] Duffy, K. J. , van Os, R. , Vos, S. , van Aarde, J. , Ellish, G. , & Stretch, A.‐M.‐B. (2002). Estimating impact of reintroduced elephant on trees in a small reserve. South African Journal of Wildlife Research, 32(1), 23–29.

[ece37465-bib-0020] Edkins, M. T. , Kruger, L. M. , Harris, K. , & Midgley, J. J. (2008). Baobabs and elephants in Kruger National Park: Nowhere to hide. African Journal of Ecology, 46, 119–125. 10.1111/j.1365-2028.2007.00798.x

[ece37465-bib-0021] Enders, C. K. , & Bandalos, D. (2001). The relative performance of full information maximum likelihood estimation for missing data in structural equation models. Structural Equation Modeling, 8(3), 430–457. 10.1207/S15328007SEM0803_5

[ece37465-bib-0022] Fornoni, J. , Valverde, P. L. , & Nunez‐Farfan, J. (2004). Population variation in the cost and benefit of tolerance and resistance against herbivory in *Datura stramonium* . Evolution, 58(8), 1696–1704. 10.1111/j.0014-3820.2004.tb00455.x 15446424

[ece37465-bib-0023] Graham, M. D. , Douglas‐Hamilton, I. , Adams, W. M. , & Lee, P. C. (2009). The movement of African elephants in a human‐dominated land‐use mosaic. Animal Conservation, 12(5), 445–455. 10.1111/j.1469-1795.2009.00272.x

[ece37465-bib-0024] Greyling, M. D. (2004). Sex and age related distinctions in the feeding ecology of the African elephant. Doctoral dissertation, University of the Witwatersrand.

[ece37465-bib-0025] Guldemond, R. A. , Purdon, A. , & Van Aarde, R. J. (2017). A systematic review of elephant impact across Africa. PLoS ONE, 12(6), e0178935. 10.1371/journal.pone.0178935 28591179 PMC5462389

[ece37465-bib-0026] Harris, G. M. , Russell, G. J. , Van Aarde, R. I. , & Pimm, S. L. (2008). Rules of habitat use by elephants *Loxodonta africana* in southern Africa: Insights for regional management. Oryx, 42(1), 66–75. 10.1017/S0030605308000483

[ece37465-bib-0027] Hempson, G. P. , Archibald, S. , & Bond, W. J. (2015). A continent‐wide assessment of the form and intensity of large mammal herbivory in Africa. Science, 350(6264), 1056–1061. 10.1126/science.aac7978 26612946

[ece37465-bib-0028] Henley, M. D. , & Cook, R. M. (2019). The management dilemma: Removing elephants to save large trees. Koedoe, 61(1), 1–12. 10.4102/koedoe.v61i1.1564

[ece37465-bib-0029] Jacobs, O. S. , & Biggs, R. (2002). The impact of the African elephant on marula trees in the Kruger National Park. South African Journal of Wildlife Research, 32(1), 13–22.

[ece37465-bib-0030] Jacobs, S. M. , & Naiman, R. J. (2008). Large African herbivores decrease herbaceous plant biomass while increasing plant species richness in a semi‐arid savanna toposequence. Journal of Arid Environments, 72(6), 891–903. 10.1016/j.jaridenv.2007.11.015

[ece37465-bib-0031] Lamb, E. G. , Mengersen, K. , Stewart, K. J. , Attanayake, U. , & Siciliano, S. D. (2014). Spatially explicit structural equation modeling. Ecology, 95, 2434–2442. 10.1890/13-1997.1

[ece37465-bib-0032] Loarie, S. R. , Van Aarde, R. J. , & Pimm, S. L. (2009). Fences and artificial water affect African savannah elephant movement patterns. Biological Conservation, 142(12), 3086–3098. 10.1016/j.biocon.2009.08.008

[ece37465-bib-0033] MacFadyen, S. , Hui, C. , Verburg, P. H. , & Van Teeffelen, A. J. A. (2019). Spatiotemporal distribution dynamics of elephants in response to density, rainfall, rivers and fire in Kruger National Park, South Africa. Diversity and Distributions, 25(6), 880–894. 10.1111/ddi.12907

[ece37465-bib-0034] Marshal, J. P. , Rajah, A. , Parrini, F. , Henley, M. , Henley, S. R. , & Erasmus, B. F. N. (2011). Scale‐dependent selection of greenness by African elephants in the Kruger‐private reserve transboundary region, South Africa. European Journal of Wildlife Research, 57(3), 537–548. 10.1007/s10344-010-0462-1

[ece37465-bib-0035] Midgley, J. J. , Balfour, D. , & Kerley, G. I. (2005). Why do elephants damage savanna trees? South African Journal of Science, 101, 213–215.

[ece37465-bib-0036] Midgley, J. J. , Coetzee, B. W. , Tye, D. , & Kruger, L. M. (2020). Mass sterilization of a common palm species by elephants in Kruger National Park, South Africa. Scientific Reports, 10(1), 1–5. 10.1038/s41598-020-68679-8 32678201 PMC7366642

[ece37465-bib-0037] Moncrieff, G. R. , Kruger, L. M. , & Midgley, J. J. (2017). Stem mortality of *Acacia nigrescens* induced by the synergistic effects of elephants and fire in Kruger National Park, South Africa. Journal of Tropical Ecology, 24, 655–662.

[ece37465-bib-0038] Morrison, T. A. , Holdo, R. M. , & Anderson, T. M. (2016). Elephant damage, not fire or rainfall, explains mortality of overstorey trees in Serengeti. Journal of Ecology, 104, 409–414. 10.1111/1365-2745.12517

[ece37465-bib-0039] O’Connor, T. G. , Goodman, P. S. , & Clegg, B. (2007). A functional hypothesis of the threat of local extirpation of woody plant species by elephant in Africa. Biological Conservation, 136(3), 329–345. 10.1016/j.biocon.2006.12.014

[ece37465-bib-0040] Olff, H. , Ritchie, M. E. , & Prins, H. H. (2002). Global environmental controls of diversity in large herbivores. Nature, 415(6874), 901–904. 10.1038/415901a 11859367

[ece37465-bib-0041] Owen‐Smith, N. , Kerley, G. I. H. , Page, B. , Slotow, R. , & van Aarde, R. J. (2006). A scientific perspective on the management of elephants in the Kruger National Park and elsewhere. South African Journal of Science, 102, 389–394.

[ece37465-bib-0042] Pienaar, D. J. , Biggs, H. C. , Deacon, A. , Gertenbach, W. , Joubert, S. , Nel, F. , Van Rooyen, L. , & Venter, F. (1997). A revised water distribution policy for biodiversity maintenance in the Kruger National Park. Internal Report South African National Parks.

[ece37465-bib-0043] Redfern, J. , Grant, C. , Gaylard, A. , & Getz, W. (2005). Surface water availability and the management of herbivore distributions in an African savanna ecosystem. Journal of Arid Environments, 63, 406–424. 10.1016/j.jaridenv.2005.03.016

[ece37465-bib-0044] Robson, A. S. , & van Aarde, R. J. (2018). Changes in elephant conservation management promote density‐dependent habitat selection in the Kruger National Park. Animal Conservation, 21(1), 302–312. 10.1111/acv.12393

[ece37465-bib-0045] Rosseel, Y. (2012). lavaan: An R package for structural equation modeling. Journal of Statistical Software, 48, 1–36.

[ece37465-bib-0046] Shannon, G. , Druce, D. J. , Page, B. R. , Eckhardt, H. C. , Grant, R. , & Slotow, R. (2008). The utilization of large savanna trees by elephant in southern Kruger National Park. Journal of Tropical Ecology, 24, 281–289. 10.1017/S0266467408004951

[ece37465-bib-0047] Shannon, G. , Page, B. R. , Duffy, K. J. , & Slotow, R. (2006). The role of foraging behaviour in the sexual segregation of the African elephant. Oecologia, 150(2), 344–354. 10.1007/s00442-006-0521-1 16927101

[ece37465-bib-0048] Shrader, A. M. , Bell, C. , Bertolli, L. , & Ward, D. (2012). Forest or the trees: At what scale do elephants make foraging decisions? Acta Oecologica, 42, 3–10. 10.1016/j.actao.2011.09.009

[ece37465-bib-0049] Smit, I. P. J. , & Ferreira, S. M. (2010). Management intervention affects river‐bound spatial dynamics of elephants. Biological Conservation, 143, 2172–2181. 10.1016/j.biocon.2010.06.001

[ece37465-bib-0050] Smit, I. P. J. , Grant, C. C. , & Whyte, I. J. (2007). Landscape‐scale sexual segregation in the dry season distribution and resource utilization of elephants in Kruger National Park, South Africa. Diversity and Distributions, 13(2), 225–236. 10.1111/j.1472-4642.2007.00318.x

[ece37465-bib-0051] Smit, I. P. J. , Peel, M. J. S. , Ferreira, S. M. , Greaver, C. , & Pienaar, D. J. (2020). Megaherbivore response to droughts under different management regimes: Lessons from a large African savanna. African Journal of Range & Forage Science, 37(1), 65–80. 10.2989/10220119.2019.1700161

[ece37465-bib-0052] Staver, A. C. (2018). Prediction and scale in savanna ecosystems. New Phytologist, 219(1), 52–57. 10.1111/nph.14829 29027662

[ece37465-bib-0053] Staver, A. C. , Bond, W. J. , Cramer, M. D. , & Wakeling, J. L. (2012). Top‐down determinants of niche structure and adaptation among African Acacias. Ecology Letters, 15(7), 673–679. 10.1111/j.1461-0248.2012.01784.x 22507561

[ece37465-bib-0054] Staver, A. C. , Botha, J. , & Hedin, L. (2017). Soils and fire jointly determine vegetation structure in an African savanna. New Phytologist, 216(4), 1151–1160. 10.1111/nph.14738 28840610

[ece37465-bib-0055] Staver, A. C. , Wigley‐Coetsee, C. , & Botha, J. (2019). Grazer movements exacerbate grass declines during drought in an African savanna. Journal of Ecology, 107(3), 1482–1491. 10.1111/1365-2745.13106

[ece37465-bib-0056] Van Coller, H. , Siebert, F. , & Siebert, S. J. (2013). Herbaceous species diversity patterns across various treatments of herbivory and fire along the sodic zone of the Nkuhlu exclosures, Kruger National Park. Koedoe, 55(1), 1–6. 10.4102/koedoe.v55i1.1112

[ece37465-bib-0057] Van Wyk, P. , & Fairall, N. (1969). The influence of the African Elephant on the vegetation of the Kruger National Park. Koedoe, 12, 57–89.

[ece37465-bib-0058] Vanak, A. T. , Shannon, G. , Thaker, M. , Page, B. , Grant, R. , & Slotow, R. (2012). Biocomplexity in large tree mortality: Interactions between elephant, fire and landscape in an African savanna. Ecography, 35, 315–321. 10.1111/j.1600-0587.2011.07213.x

[ece37465-bib-0059] Venter, F. J. (1990). A classification of land for management planning in the Kruger National Park (pp. 1–394). PhD thesis, University of South Africa (UNISA), South Africa.

[ece37465-bib-0060] Wakeling, J. L. , Staver, A. C. , & Bond, W. J. (2011). Simply the best: The transition of savanna saplings to trees. Oikos, 120(10), 1448–1451. 10.1111/j.1600-0706.2011.19957.x

[ece37465-bib-0061] Więski, K. , & Pennings, S. (2014). Latitudinal variation in resistance and tolerance to herbivory of a salt marsh shrub. Ecography, 37(8), 763–769. 10.1111/ecog.00498

[ece37465-bib-0062] Wigley, B. J. , Coetsee, C. , Kruger, L. M. , Ratnam, J. , & Sankaran, M. (2019). Ants, fire, and bark traits affect how African savanna trees recover following damage. Biotropica, 51(5), 682–691. 10.1111/btp.12683

[ece37465-bib-0063] Williams, H. F. , Bartholomew, D. C. , Amakobe, B. , & Githiru, M. (2018). Environmental factors affecting the distribution of African elephants in the Kasigau wildlife corridor, SE Kenya. African Journal of Ecology, 56, 244–253. 10.1111/aje.12442

