# Supplementary material for: Heterogeneity in African savanna elephant distributions and their impacts on trees in Kruger National Park, South Africa
Source: Ecol Evol. 2021 Apr 6;11(10):5624–34. doi: 10.1002/ece3.7465 (PMC8131780; doi:10.1002/ece3.7465)
Supplement: Supplementary file 1 — Supplementary Material [file ECE3-11-5624-s001.docx]

**SUPPLEMENTARY INFORMATION**

**TABLES**

**Table S1.** Correlation matrix for all variables included in structural equation model. ‘Geology’ = coded such that positive relationships are biased towards basalt sites, and negative ones toward granite; ‘rain’ = mean annual rainfall; ‘river’ = distance to the nearest permanent river; ‘waterpoint’ = distance to nearest artificial waterpoint open in 2011; ‘tree dens’ = tree density; ‘grass’ = grass biomass; ‘damaged stem’ = density of elephant-damaged stems; ‘bulls’ = elephant density, bulls alone or in small groups; ‘herds’ = elephant density, herd members; and ‘impact’ = percentage of trees damaged by elephants.

|  | geology | rain | river | waterpoint | tree dens | damaged stem | | grass | bulls | herds |
| --- | --- | --- | --- | --- | --- | --- | --- | --- | --- | --- |
| rain | -0.37 | 1 |  |  |  | |  |  |  |  |
| river | 0.13 | -0.13 | 1 |  |  | |  |  |  |  |
| waterpoint | -0.03 | -0.16 | 0.11 | 1 |  | |  |  |  |  |
| tree dens | -0.38 | -0.10 | 0.02 | 0.00 | 1 | |  |  |  |  |
| damaged stem | -0.03 | -0.37 | 0.12 | 0.11 | 0.32 | | 1 |  |  |  |
| grass | 0.20 | 0.47 | 0.07 | -0.07 | -0.36 | | -0.21 | 1 |  |  |
| bulls | 0.30 | -0.17 | -0.04 | -0.19 | -0.31 | | -0.07 | 0.08 | 1 |  |
| herds | -0.20 | -0.03 | -0.50 | -0.01 | 0.01 | | 0.04 | -0.28 | 0.08 | 1 |
| impact | 0.04 | -0.20 | -0.03 | -0.04 | 0.30 | | -0.02 | -0.20 | 0.04 | 0.03 |

**Table S2.** Results of structural equation modeling of relationships among savanna vegetation and elephants in Kruger National Park, South Africa. Candidate models were constructed by removing suspect links one at a time; top models shown here, with variables that differ between candidate models underlined for emphasis.*

| Model Structure | | *ΔAICc* | *p* from *χ^2^* |
| --- | --- | --- | --- |
| impact ~ geology + tree dens + grass + bulls  bulls ~ geology + rain + waterpoint + tree dens  herds ~ geology + river + tree dens + grass + damaged stem | tree dens ~ geology + rain  damaged stem ~ geology + rain + river  grass ~ geology + rain + river  river ~ rain | **0** | 0.002 |
| impact ~ geology + tree dens + grass  bulls ~ geology + rain + waterpoint + tree dens  herds ~ geology + river + tree dens + grass + damaged stem | tree dens ~ geology + rain  damaged stem ~ geology + rain + river  grass ~ geology + rain + river  river ~ rain | 0.65 | 0.002 |
| impact ~ geology + tree dens + grass + bulls  bulls ~ geology + rain + waterpoint + tree dens  herds ~ geology + river + tree dens + grass + damaged stem | tree dens ~ geology + rain  damaged stem ~ geology + rain  grass ~ geology + rain + river  river ~ rain | 0.96 | 0.001 |

*Note that large *p*-values are desirable in structural equation modeling.

**Table S3**. Results of binomial testing by tree species for all trees with more than fifteen observed individuals. *P*-value gives confidence that calculated mean differs from the across-species average damage rate of 28.0%. Significant results are bolded.

|  |  |  | 95% confidence interval | |  |
| --- | --- | --- | --- | --- | --- |
| Species | *n* | Mean | Low | High | *p* |
| ***Sclerocarya birrea*** | **29** | **0.55** | **0.36** | **0.74** | **<0.01** |
| ***Acacia tortilis*** | **30** | **0.53** | **0.34** | **0.72** | **<0.01** |
| *Acacia gerrardii* | 20 | 0.45 | 0.23 | 0.68 | 0.13 |
| ***Combretum zeyheri*** | **111** | **0.42** | **0.33** | **0.52** | **<0.01** |
| *Albizia harveyi* | 15 | 0.40 | 0.16 | 0.68 | 0.39 |
| ***Colophospermum mopane*** | **1,081** | **0.39** | **0.36** | **0.42** | **<0.001** |
| *Acacia welwitschii* | 29 | 0.38 | 0.21 | 0.58 | 0.30 |
| ***Combretum apiculatum*** | **770** | **0.36** | **0.33** | **0.40** | **<0.01** |
| *Peltophorum africanum* | 17 | 0.35 | 0.14 | 0.62 | 0.59 |
| *Acacia nigrescens* | 114 | 0.34 | 0.26 | 0.44 | 0.14 |
| *Combretum imberbe* | 33 | 0.33 | 0.18 | 0.52 | 0.56 |
| *Philenoptera violacea* | 37 | 0.30 | 0.16 | 0.47 | 0.85 |
| *Combretum hereroense* | 64 | 0.28 | 0.18 | 0.41 | 1.00 |
| *Terminalia prunioides* | 73 | 0.21 | 0.12 | 0.32 | 0.19 |
| ***Dichrostachys cineria*** | **151** | **0.19** | **0.13** | **0.26** | **<0.01** |
| ***Terminalia sericea*** | **187** | **0.13** | **0.09** | **0.19** | **<0.001** |
| ***Acacia exuvialis*** | **47** | **0.11** | **0.04** | **0.23** | **<0.01** |
| ***Euclea divinorum*** | **28** | **0.07** | **0.01** | **0.24** | **<0.05** |


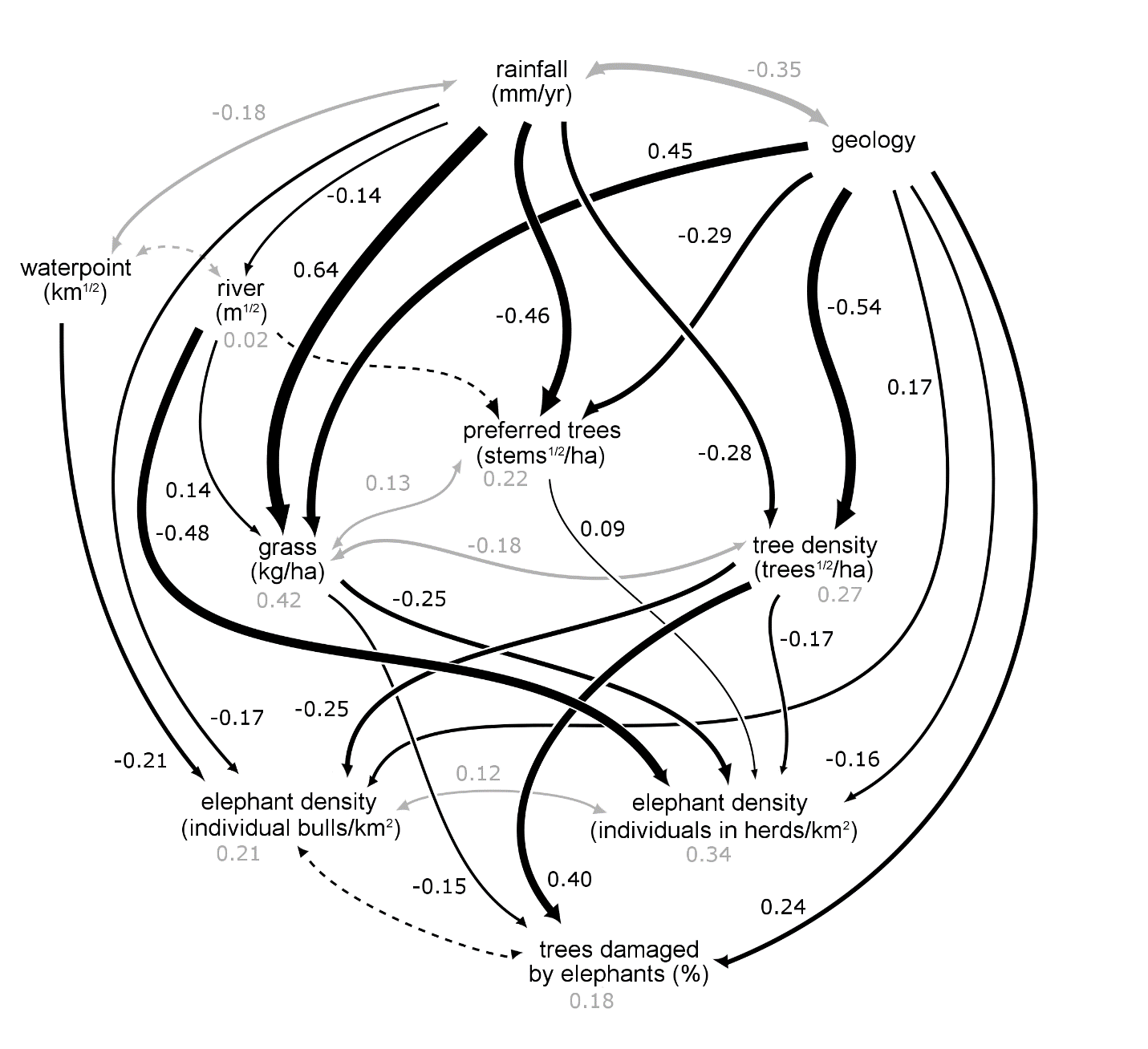


**Figure S2.** Structural equation modeling results showing probable interactions among water, geology, vegetation, and elephants in Kruger National Park, South Africa. Solid lines show supported effects; dashed lines show effects that are probable, but locally insignificant; and line widths scale with the strength of the effect. Black single-headed arrows indicate an assumed causal effect, while gray double-headed arrows make no assumption about causation. Grey text attached to each dependent variable gives its total R^2^. ‘Geology’ is coded such that positive relationships are biased towards basalt sites, and negative ones toward granite. See Tables S1-S2 for model selection details.


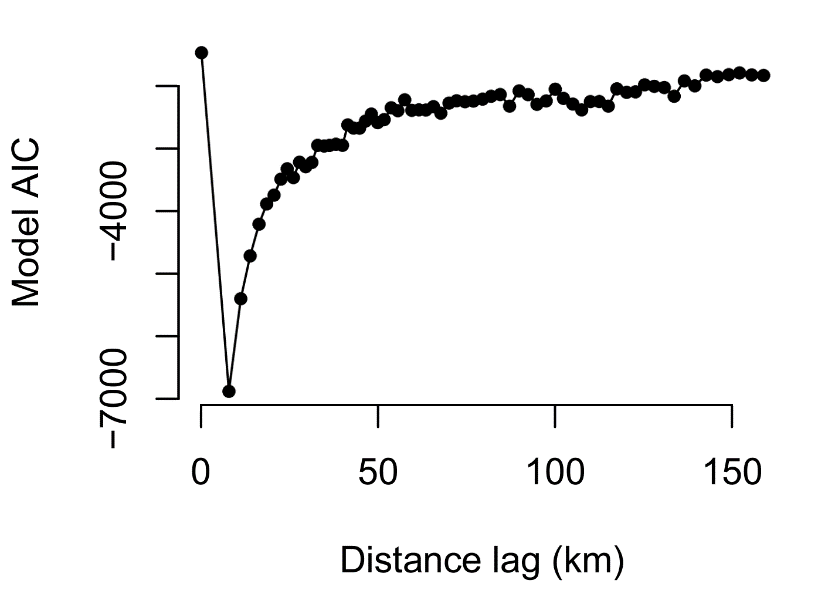


**Figure S3.** Effect of lag distance on model AIC in spatially explicit structural equation modeling (SESEM). Models with short lags are preferred over those with either no lag or long lags.


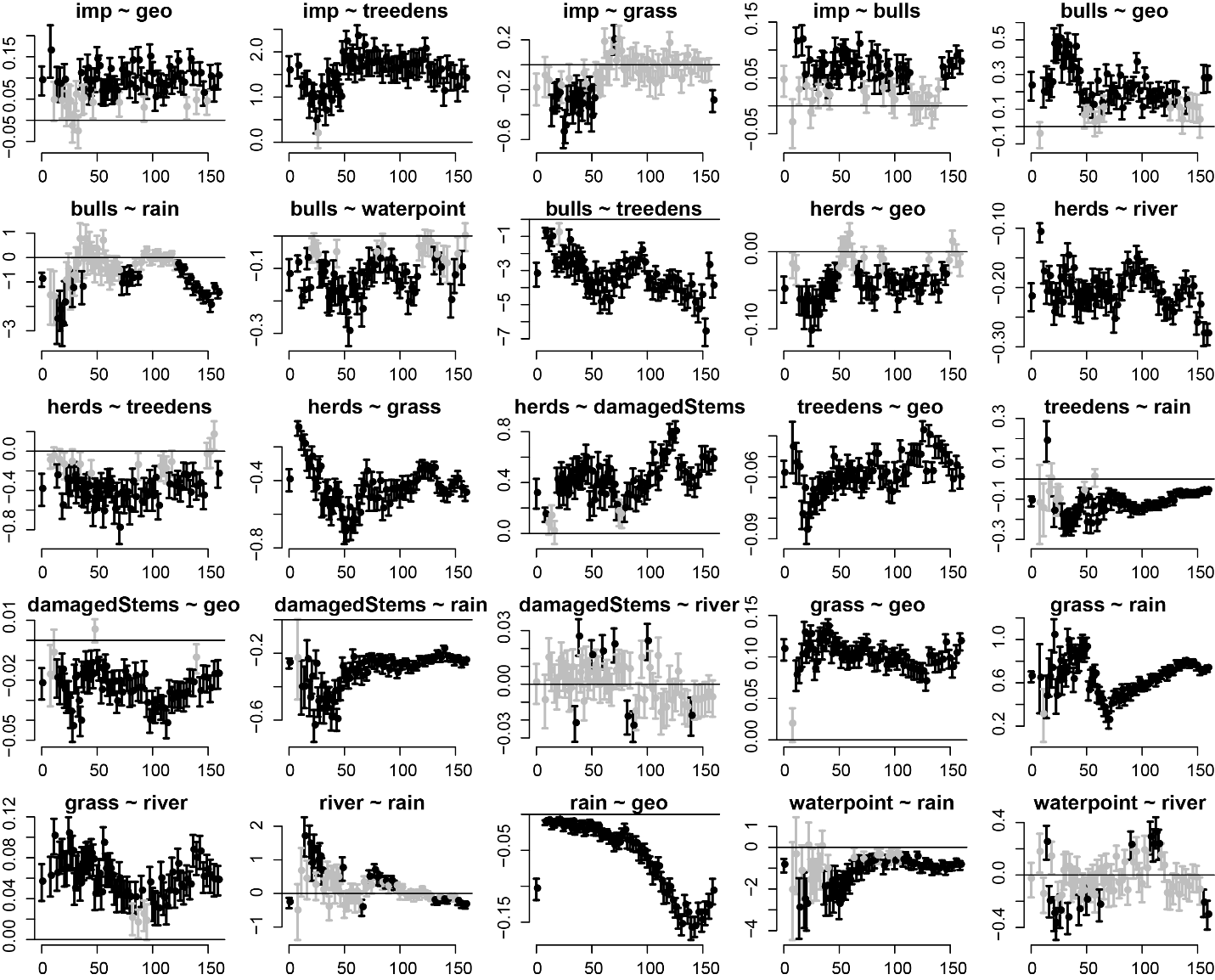


**Figure S4.** Effect of lag distance on path coefficients for SESEM. Elephant damage to trees is concentrated in densely treed areas regardless of lag distance, and preferentially damaged trees are consistently concentrated in dry, granitic areas. Whereas bull density is most influenced by tree density, herd density is instead correlated with grass biomass and river proximity.
